# Computer simulations reveal changes in the conformational space of the transcriptional regulator MosR upon the formation of a disulphide bond and in the collective motions that regulate its DNA-binding affinity

**DOI:** 10.1371/journal.pone.0192826

**Published:** 2018-02-22

**Authors:** Amanda Souza Câmara, Eduardo Horjales

**Affiliations:** Departamento de Física e ciência interdisciplinar, Instituto de Física de São Carlos; Universidade de São Paulo, São Carlos, SP, Brasil; Bioinformatics Institute, SINGAPORE

## Abstract

*M*. *tuberculosis* oxidation sense Regulator (MosR) is a transcriptional regulator from *Mycobacterium tuberculosis*. It senses the environment oxidation and regulates the expression of a secreted oxidoreductase, thus defending the bacilli against oxidative stress from the phagosome. While most of the members of the Multiple antibiotics resistance Regulator (MarR) family are ligand-responsive, MosR may dissociate from its DNA site upon formation of an intrachain disulphide bond. However, the structure of MosR in its oxidized state is not known, and it is not clear how the formation of this disulphide bond would lead to the conformational changes required for dissociation of the DNA. Nonetheless, MosR presents two crystallographically resolved conformations in its reduced state: bound and unbound to DNA. We managed to simulate MosR unbound to the DNA, both in the presence and in the absence of the disulphide bond. Our results indicate that this disulphide bond precludes the N-terminal residues from adopting a conformation that stands in-between the helix α1 and the DNA binding domain (DBD) from the other chain. Once this conformation is achieved in the reduced state, this DBD detaches from the dimerization domain and becomes more flexible, being able to perform motions with higher amplitude and higher degree of collectivity. Only then, MosR may achieve a conformation where its recognition helices fit into the major grooves of its DNA site. The analysis of the collective motions performed by MosR, during the different situations sampled by the molecular dynamics (MDs), was only possible by the method of filtering harmonic modes with specific frequencies. The frequency of the collective motions performed by the DBD of MosR in the reduced state to achieve a DNA-binding conformation is in the range of 20 to 50 MHz, but it may be associated to more sporadic events since it requires the combination of a suitable conformation of the N-terminal residues.

## Introduction

*Mycobacterium tuberculosis* is the causative agent for tuberculosis. Once it reaches the lungs of its host, it is phagocytized by macrophage cells as a defense mechanism of our body in response to the exogenous agent. Inside the phagosome the bacilli thrive in a very harmful environment. While the macrophage is trying to lysate it, it releases several defenses which prevent the maturation process needed for the phagosome to fuse with the lysosome. As long as the immune system of its hostage is not injured, the bacilli may live there for several years, without ever causing tuberculosis symptoms to appear [1].

To prosper inside the phagosome, *M*. *tuberculosis* needed to evolve a very sensitive perception of its surroundings, and also a prompt response to any perturbation. *M*. *tuberculosis* oxidation sense Regulator (MosR) plays an indispensable role for *M*. *tuberculosis* prosperity in the phagosome. MosR is a transcriptional repressor that senses the environment oxidation and regulates the expression of a secreted oxidoreductase, thus affecting the pH in the phagosome interior. Hence its name, *M*. *tuberculosis* oxidation sense Regulator (MosR) [2].

Thus, MosR is characterized into the Multiple antibiotics resistance Regulator (MarR) family. This family includes critical proteins for controlling of bacterial responses to oxidative stresses and antibiotics, control of virulence factors, as well as production and catabolism of environmental aromatic compounds [3]. Proteins of this family share structural similarities that are also present in MosR. They are homodimers of triangular shape, presenting a dimerization domain which connects to two DNA-binding domains (DBD). The dimerization is stabilized mainly by hydrophobic contacts between intercalating N- and C-terminal α-helices. In particular, the long helices α1 and α5 are the connection to the DBD, described as a winged helix motif [3,4]. Proteins of this family may also contain ligand binding sites that reside in the DBD where the ligand appears as a competitive effector or in between the dimerization domain and the DBD. In the latter case the ligand participates in an allosteric mechanism that promotes large conformational changes to the DNA binding site [3,5].

The crystallographic structure of MosR has already been elucidated in its reduced conformation, both bound and unbound to the DNA [2]. An effector site is described to lie in the dimerization domain with two cysteine residues susceptible to the formation of a reversible disulphide bond [2]. These two cysteines are important for the environment oxidation sensing mechanism, as concluded by experiments performed by Brugarolas and collaborators [2]. Using electrophoretic mobility shift assays of MosR with DNA upon increasing concentrations of oxidants, they have detected the dissociation of the protein upon the addition of 0.5–1 mM of oxidant [2]. Furthermore, by *in vivo* RT-PCR assays they were able to monitor the levels of the oxidoreductase’s mRNA and concluded that it was increased upon addition of H_2_O_2_. Moreover, punctual mutation of one of the cysteine residues combined with an increased concentration of oxidants allowed them to identify partial dissociation of the MosR from the DNA, and lower levels of expression of the oxidoreductase’s mRNA which might indicate the presence of additional oxidation-sensing mechanisms. The authors concluded that MosR has greater affinity for its specific DNA site in its reduced form, while in its oxidized form it is not likely for binding, allowing the oxidoreductase to be expressed [2].

Although the structure of MosR on its reduced state has been described, the structure of its oxidized state remains unknown and it is still not clear how the disulphide bond formation would lead the protein molecule to dissociate from its DNA site.

The Organic hydroperoxide resistance Repressor (OhrR), is a protein homologous to MosR (28% of amino acid sequence identity when MosR sequence is compared to OhrR from *B*. *subtilis* and 25% identical compared to OhrR from *X*. *campestris*). OhrR is also sensitive to oxidation and its molecular structure was elucidated in its oxidized state (from *Xanthomonas campestris*) [6]. Both proteins may display a reversible disulphide bond, changing from an oxidized to a reduced state. During this reversible process, one cysteine residue is in a conserved position surrounded by aromatic amino acids (typically found in oxidation-sensing regulators [7]).

Experimental results for both proteins indicate that this specific reversible bond may alter the affinity of the protein to its DNA site. In fact, the oxidized structure of OhrR indicates a rotation of the recognition helix α4 when compared to the reduced conformation, which prevents the protein from binding to its DNA site when oxidized [6]. This rotation arises when Cys22 (located in the N-terminal of helix α1) forms a disulphide bond with Cys127’ (located in the long helix α5’ which connects the dimerization domain to the DBD). This strong interaction causes helix α5’ to break into two smaller helices, leading to a different orientation of the DBD.

However a striking difference between MosR and OhrR lies in which residue is capable of forming the disulphide bond. While in OhrR the residues are the Cys22 from one chain and the Cys127’ from the other, holding close two different helices, α1 and α5’, and symmetrically, Cys22’ from α1’ and Cys127 from α5; for MosR the bond is formed between Cys10 and Cys12 from the same chain and same helix α1, and symmetrically Cys10’ and Cys12’ in helix α1’. Therefore, it is not likely that the disulphide bonds in MosR would promote a break in the long helices α5 and α5’ as in OhrR. Nevertheless, a rotation of helices α4 and α4’ is also observed in MosR by comparison of its two resolved structures with two different states: bound and unbound to DNA.

By comparing the two crystallographic structures, it is possible to observe major differences on the DBD. It is also observed that helix α4 which fits into the major groove of the DNA helix, is rotated for about 25° from one conformation to the other, which is similar to OhrR upon the formation of the disulphide bond.

The described differences are observed albeit the crystallographic structures are both at the reduced state (either bound or unbound to the DNA). Nevertheless, the reduced state of MosR is also the active state, which unbound to the DNA may be dynamically changing between different conformational populations. Thus, it is likely that the structure of MosR reduced and unbound to DNA–a temporal average of all the visited conformations—resembles its non-binding oxidized structure. An analysis of the different features between bound and unbound conformations may bring insights on the oxidized conformation and how the changes caused by the disulphide bond may lead to dissociation from the DNA.

In both MosR structures some N-terminal residues are disordered and thus, not included in the crystallographic structure. In the structure unbound to the DNA there are three residues present that are disordered in the bound structure, indicating greater flexibility of this region when the protein is bound to the DNA. Interestingly, the N-terminus loop resides in a key position, it is not only closely followed in sequence by the two cysteines that may form the disulphide bond, but it is also located in a region responsible for great conformational changes in the protein, in-between helix α5 and the DBD. However, neither of the resolved structures presents the coordinates for the entire N-terminus. Indeed, five residues are missing in the unbound conformation and the bound conformation lacks eight residues. This short but rather flexible disordered region may interact with helix α5 as well as with helices α2 and α3 from the DBD, and it may be affecting the motions of this domain.

The greater flexibility of the DNA binding domain is due to the rigid body movement with respect to the dimerization domain [3]. This is essential for MosR to adopt different conformations as a mechanism to regulate its affinity for the DNA. Some conformations would be more likely of binding to the DNA than others because the geometry of two consecutive major grooves on the DNA double helix structure requires the DNA recognition α-helices of both monomers in the MosR dimer to adopt a particular orientation [8]. Therefore, the disulphide bond should prevent MosR from reaching conformations of greater affinity to its DNA site. This may be done by decreasing the flexibility of the DNA binding domain and holding the protein molecule in a conformation where the recognition α-helices do not present a most favorable orientation [9].

A protein conformation is favorable for binding when the α-helices responsible for the DNA recognition have the same orientation as the two consecutive major grooves. Thus, the relative orientation of both recognition α-helices determines the ability of the protein to bind to the DNA. Once the recognition residues interact with specific bases in the DNA grooves, a fine tune of the DNA binding can be done by the rest of the residues and the α-helix main chain can adopt a better fit orientation [10]. Thus, the binding of the protein to its DNA site requires both collective and local conformational changes: collective changes that allow both α-helices to be simultaneously closer to two consecutive major grooves; and local deviations to fine tune the binding.

These collective and local conformational changes differ from each other in the number of atoms involved and the total inertial mass of this group of atoms subjected to the same collective motion. Collective motions present low frequencies while local motions show high frequencies.

The low frequency motions involve residues from the same region moving as a rigid body, while the high frequency motions are characteristic from side chain rotations or even single bond vibrations [11,12]. Thus, it takes longer times to approximate the α-helices than to specifically fit their orientation.

The disulphide bond in MosR is located away from the DBD. For this reason, the disulphide bond must affect a motion that connects its own site to the DNA-binding site in order to promote a change in the DNA-binding-affinity. Thus, the disulphide bond probably affects some of the low frequency motions, which connect large groups of atoms and may prevent the recognition α-helices to approximate to an adequate orientation. In fact, many studies have emphasized how important a broad conformational ensemble can be for the biological function of the protein.

A recent work highlights the importance of the internal motions of the proteins for regulating its activity [13]. Using NMR spectroscopy experiments and thermodynamic data, the authors described a clear relationship between the internal dynamics of the catabolite activator protein (fast and slow motions) and its stability to bind to the DNA.

Few experiments are able to detect the low frequency motions of the proteins. Relaxation times are measured by NMR spectroscopy or laser Raman spectroscopy. TLS parameters and anisotropic displacements are obtained from crystallographic structure determination and also contain information on the collective (low frequency) motions.

In the present study, we use a computational approach by simulating MosR in hundreds of ns long molecular dynamics in order to detect how the formation of a disulphide bond could affect the accessible conformations during thermal motions of the protein and its affinity for the DNA.

Because the N-terminal region may affect the flexibility of the DBD, we have started our simulations with a set of possible positions for this region, which has not been solved in the crystallographic structures determinations. A detailed analysis of the interactions that the protein might form in the presence and in the absence of the disulphide bonds is presented here.

Moreover, because the biological conformational changes are related to low frequency motions, we focused on retrieving these motions from molecular dynamics simulations of both states of MosR: reduced and oxidized. This is achieved using an analysis based on the Fourier filter transform proposed by Oguthorpe in the late ‘90s [14,15]. The time intervals of simulation have become much larger since then and new outcomes can be withdrawn by the application of this methodology. Another advantage of this tool is that it makes possible to observe the collective motions that appear in a simulated trajectory, while relating them to the frequency they occur with.

## Materials and methods

### The molecular dynamics simulations

All simulations were carried out using Gromacs program [16] and the united-atom force field Gromos54a7 [17]. Solvation of the system was performed using SPC/E model [18] for water molecules and 16 chlorine counter ions were added in a box whose sides are minimum 1 nm away from a protein atom.

The original structure of MosR used in the simulations was the apo crystallographic conformation (unbound to DNA) solved by Brugarolas and collaborators (PDB entry 4fx0) [2]. This structure presents missing residues mainly in chain A, hence a starting conformation for the simulations was built using a copy of chain B superposed to the original chain A. Residues 1 to 5, 51 to 53, 92 to 95 and 148 were previously added to chain B using Coot program [19] and applying the regularize zone routine. Additionally, 12 different positions of the N-terminal residues were tested. Each conformation was minimized for 1,000 steps using steepest descent algorithm. Equilibration was performed in three steps, an initial equilibration was executed for 100 ps restraining the position of all protein atoms in a canonical ensemble at 310 K, followed by another equilibration step for 100 ps still restraining the protein but in an isobaric-isothermal ensemble at 1 bar and a third equilibration step for 1 ns in this same ensemble allowing the N-termini (residues from 1 to 7) and the solvent to move and the system was gradually heated from 0 to 310 K during 500 ps. All three equilibration steps were calculated using periodic boundary conditions, LINCS algorithm to constraint the hydrogen bonds [20], PME algorithm to calculate electrostatic forces [21], velocity rescaling algorithm on the temperature control [22] and Parrinello-Rahman on the pressure control when necessary [23] and the steps were 2 fs long.

Two of these conformations were chosen, because of their higher stability, to proceed with a 1 ns long step of equilibration allowing the entire protein to move in an isobaric-isothermal ensemble after been gradually heated from 0 to 310 K during 500 ps. A 400 ns long molecular dynamics was then simulated for each one, saving conformations at each 10 ps.

These two equilibrated conformations were transformed to build the oxidized state of each conformation. Thus, we manually chose another rotamer for each Cys12, bringing the sulphur atom closer to the one in Cys10. After defining the new S-S bond, we applied the regularize zone routine from the Coot program. A new topology for these structures was then built, which constitute the oxidized state of MosR. These two oxidized conformations were minimized and equilibrated following the same protocol used with the original reduced conformations (except for the N-terminal equilibration). Then, a 400 ns long molecular dynamics was simulated for each one, saving conformations at each 10 ps to analyse the differences generated by both initial N-terminal conformations.

A third conformation was chosen to simulate a 100 ns long molecular dynamics using a distance restraint for the two pair of atoms consisting of the nitrogen atom from Ala6 and the oxygen atom from Arg65 of the opposite chain. This restriction was imposed by setting the parameters low, up1 and up2 with the values of 0.24 nm, 0.4 nm and 0.5 nm, respectively. Minimization and equilibration were also calculated using these restraints, besides the above mentioned default conditions, as well as the molecular dynamics simulation, from which a conformation was saved at each 10 ps.

We encourage the interested readers to contact the authors asking for the trajectory files. The trajectory files of the five performed simulations, limited to only the protein atoms coordinates, may be found in figshare.com public repository in dcd format.

### Analyses

The results here presented are based on three different analyses. Own algorithms were written in Fortran 90 to perform the different analyses.

The first group of calculations was implemented based on the subroutines from Martínez L. [24] in order to read and write the trajectory files in dcd format. Furthermore, recipes from [25] were used to calculate the Fourier transform of the simulated trajectories and the principal inertia axes of the α-helices.

Thus, we could determine the evolution of geometrical quantities of the protein structure during the simulated trajectory, such as distances between chosen atoms or centers of mass, principal axes of α-helices, and the angle between them. These geometrical quantities allowed us to characterize the orientation of the recognition helices in the DNA-binding domain, which are described in detail in the S1 Appendix. Moreover, contacts between residues have been accounted to analyze interactions with the N-terminal. A contact was considered to exist when two α-carbons were not more than 6 Å apart.

The second group of calculations determined the isotropic and anisotropic displacements as defined in [26,27], from which information was obtained on the flexibility of the protein and on the direction of the motion it performs during the simulations.

The third method has been already mentioned for retrieving low-frequency motions from molecular dynamics, which we adapted from the filtering method of Osguthorpe [14–15], and it is also explained below.

To calculate the displacements and harmonic modes within the trajectory, we first superposed all conformations to a reference conformation. Unless explicitly mentioned, we performed alignments, only on the dimerization domain of the protein, specifically residues from 6 to 38 and from 111 to 147, except to calculate the RMSD of the recognition helices, which requires the superposition of only the recognition helices, defined by the residues from 69 to 83.

For visual analysis, PyMOL [28] was used in order to represent the ellipses of the anisotropic atomic displacements and VMD [29] for the representation of the atomic displacement vectors by arrows.

#### The filtering analysis

The use of Fourier transform to filter frequencies is common in signal analysis [25,30]. This is usually applied to remove noisy frequencies, in a variety of data processing procedures as time series analysis or image processing. As showed in [14,31], the mentioned method can be performed to filter any time dependent parameter extracted from a molecular dynamics, including the 3N coordinates from the protein trajectory (where N is the number of atoms of the protein). This last application results in a filtered trajectory, in which high or low frequency motions can be neglected, allowing the analysis of the protein motion in certain frequencies of interest.

In [15] this filtering method is used to extract harmonic motions, selecting one specific frequency, and thus comparing to the modes obtained by Normal Mode Analysis. However, this process maintains the asymmetry present in the simulated trajectories and it results on different phases for each coordinate. For the present study, it was preferred to generate a new function of time, with twice the time interval of the simulated trajectory, calculating the symmetric function around the origin. This new function still obeys the Newton’s equations of motion. Then, the Fourier transform allowed us to select only one frequency and designate it to a single phase and amplitude in a harmonic motion for the entire protein, as in Eq (1),

$${\vec{\Delta x}}_{n}=S_{n}{\hat{w}}_{n}\sin\left(2\pi \omega_{n}t\right)$$ (1)

where the sub-index *n* stands for each calculated mode, *ŵ*_*n*_ is a versor that represents the direction of each harmonic motion of the protein in the 3N space, *S_n_* is the squared amplitude of this motion, *ω*_*n*_, its frequency, and ${\vec{\Delta x}}_{n}$ is the displacement vector of the entire protein in each harmonic mode. A more detailed explanation lies on the S2 Appendix; a summary of the method consisting of five steps is listed below:

1. Make the initial and final values of the trajectory function equal zero by subtracting a linear function of time that starts at the initial position and ends at the final position of the trajectory.
2. Take the sine transform: This is equivalent to the Fourier transform of a trajectory with a double time period (from–T to T) and anti-symmetric in time.
3. Filter one or a range of frequencies;
4. Transform back to the time domain;
5. Add the linear function subtracted in step 1.

This filtering can be calculated over any property of the molecular dynamics that is a function of time. However, when filtering the trajectory of a molecule these steps should be applied over all of its 3N coordinates separately, resulting in unidirectional motions of the entire protein in the 3N space, which may be represented by atomic displacement vectors for each atom of the protein similarly to Normal Mode Analysis (NMA) or Principal Component Analysis (PCA).

An advantage of this method is that it directly relates frequencies to amplitudes of harmonic motions retrieved from the molecular dynamics trajectory, which is simulated in specific conditions of temperature, pressure and constraints. This fills a blank left by PCA, which does not carry time information and depends on other procedures to recover it, and also gets around a misleading result from NMA, which incorrectly approximates the broad potential energy wells to the narrow wells present at the bottom of the potential energy landscape [32]. However, the harmonic modes calculated by the Sine Filtering Analysis here we proposed, does not form an orthonormal basis. The method actually provides as many modes as the number of given conformations. The range of possible frequencies it may analyze is restricted to the time window of the trajectory, as in Eq 2,

$$\omega_{n}=\frac{n}{2\delta tK},\;n=0,1,\ldots ,K$$ (2)

where *ω*_*n*_ is the frequency, *n* is the mode number, *K* is the total number of conformations and *δt* is the time step between each conformation.

Nevertheless, they may be quantitatively analyzed and compared not only by the associated frequency and amplitude, but also by dot products to other harmonic modes that indicates the similarity on the direction of the motion, providing complementary information to the other mentioned methods.

In this study we only compared modes obtained by the mentioned filtering method. We compared them by their frequencies, amplitudes and degree of collectivity. The amplitude was obtained from Eq 1, and it was calculated for any set of atoms of interest. We considered all the α-carbon atoms for the global amplitude, and only the α-carbon atoms from the recognition helices for the local amplitude of the recognition helices. The degree of collectivity *κ*_*n*_ was considered as suggested by Tama and Sanejouand [33] and it was calculated as in Eq 3, where *Δr*_*in*_ is the displacement of each atom *i* in the mode *n*, *S*_*n*_ is the squared amplitude of the atomic displacement and *N* is the total number of atoms, which we considered to be all α-carbon atoms.

$$\kappa_{n}=\frac{1}{N}e^{-\sum_{i=1}^{N}S_{n}{\left(\Delta r_{in}\right)}^{2}\log\left[S_{n}{\left(\Delta r_{in}\right)}^{2}\right]}$$ (3)

## Results and discussion

The results of the four simulations are showed below. Each simulation is 400 ns long. These four simulations differ from each other in the initial position of the N-termini or in the oxidation state of the protein. In this study, the oxidation state of the protein is defined as dependent of the existence of two intrachain disulphide bonds built, as explained earlier between residues Cys10 and Cys12. In the oxidized state, both disulphide bonds are present, while in the reduced state they are not. Simulations 1 and 2 are in the reduced state, while simulations 3 and 4 are in the oxidized state. Furthermore, simulations 1 and 3 share the same initial conformation of the N-terminal aminoacids, whereas 2 and 4 share another initial conformation of this region.

The following results attempt to characterize the similarities and the differences of the apo MosR in its oxidized or reduced states. Furthermore, it also describes how this distinct features affect the affinity of MosR for the DNA site.

**Fig 1:**
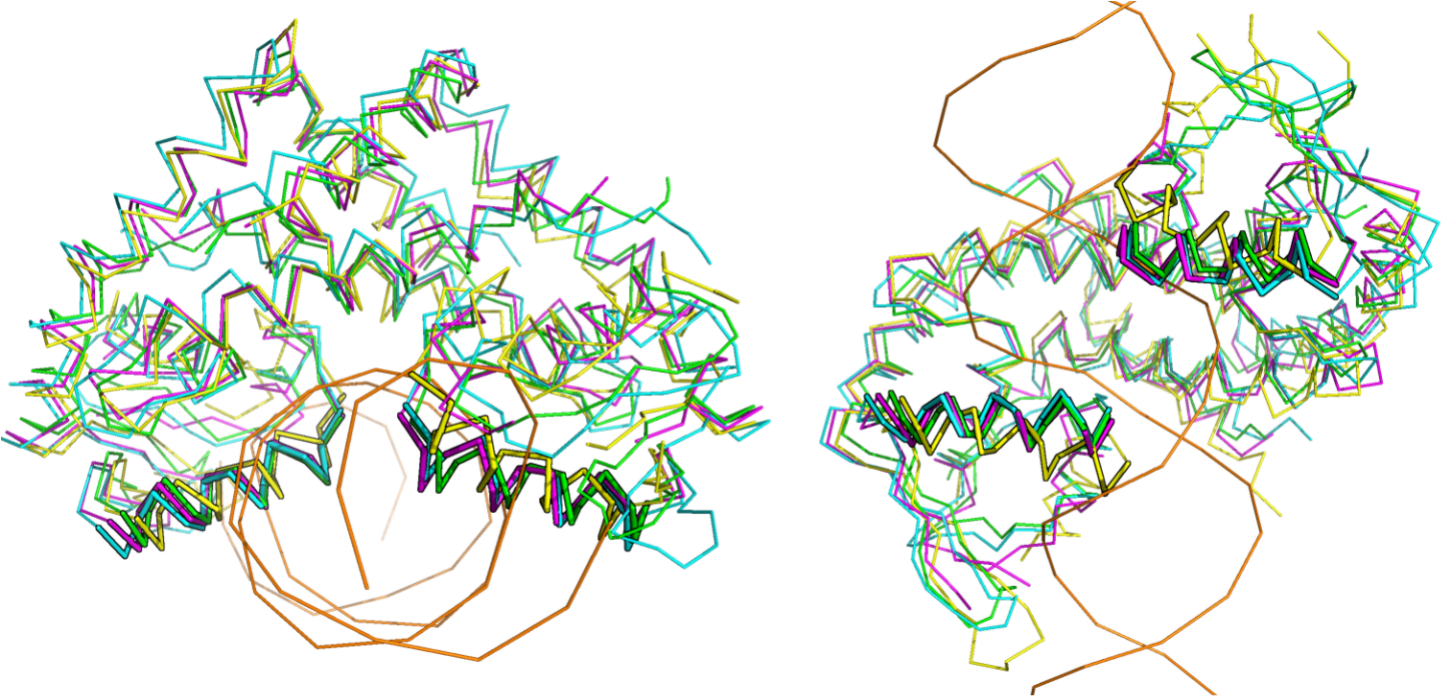
Superposition of four conformations of MosR, in two different orientations. The positions of the recognition helices are indicated by thicker ribbons. The average conformation of the two molecular dynamics (800 ns) in the reduced state is colored in green, the average conformation of the two molecular dynamics (800 ns) in the oxidized state is colored in blue, the apo crystallographic structure (PDB entry 4fx0) is colored in pink and the holo crystallographic structure (PDB entry 4fx4) is colored in yellow with its DNA in orange.

Fig 1 shows both simulated states of MosR, calculated as the average conformations of simulations 1 and 2 for the reduced state, and 3 and 4 for the oxidized state superposed to the apo and holo crystallographic structures.

Table 1 indicates the RMSD of each conformation (average oxidized and average reduced) to the other and to both known crystallographic structures (apo and holo reduced structures). The RMSD considering only the recognition helices is also listed for each pair of conformations. Although both conformations are simulated in different states, both have a lower RMSD when comparing each one to the apo structure than when the RMSD is calculated in relation to the holo structure. This is even more prominent when the RMSD is calculated only on the α-carbon atoms of the recognition helices.

**Table 1:** RMSD values between the apo structure, holo structure, oxidized average conformation and reduced average conformation.

	Apo structure	Holo structure	Oxidized average conformation
Reduced average conformation	1.112–0.621	1.887–3.076	1.665–0.873
Oxidized average conformation	1.369–0.693	2.684–3.214	
Holo structure	1.520–3.092		

Two RMSD values (Å-Å) are displayed for each pair of conformations separated by a hyphen. The first one is regarding all α-carbons and the second one considers only the α-carbons of the recognition helices.

An average conformation of all four simulations presents a RMSD from the apo crystallographic structure of only 1.0 Å. Nevertheless, a few differences between the oxidized state and the reduced state can be observed, which is in accordance to the differences also observed between the crystallographic structures [2]. The formation of the disulphide bond at the beginning of the helix α1 causes an extra tension at the next turn, which adopts a slightly different structure (as showed in Table 2 which indicates the distance between the α-carbon atoms of residues Cys10 and Thr14) observed in the crystallographic structures and in the calculated averages of the two simulated states. Indeed, 3_10_ helices are more likely to form intrachain disulphide bridges than α-helices [34]. For example, thioredoxin reductase presents conformational changes involving a disulphide bridge in the N-terminal of an α-helix, which is in a position similar to the cysteines in MosR [35]. In addition, a hydrophobic region appears around the disulfide bond which repels charged residues that can be present in the neighborhood. Indeed, Asn37 appears to be farther from Cys12’ in the simulated oxidized state and in the apo crystallographic structure than in the reduced state and the holo crystallographic structure (Table 2). However, a maximum rotation of only 5.9° is observed between the recognition helices of the average conformations in the oxidized and reduced state (Fig 1), which is less than the rotation of 25° observed between the recognition helices of the apo and holo crystallographic structures. Since the complex cannot be formed in the oxidized state, the next results were performed to evaluate whether the simulations can reproduce the experimentally observed difference between the two states in relation to the affinity for the DNA site. We also analyzed some other differences observed between the two crystallographic structures [2]. It is important to notice that some of them maybe caused by the interaction with the DNA, thus it is difficult to attribute them to a state of the protein, either reduced or oxidized. Nevertheless, the recognition helices are known to be the first to interact with the DNA during the complex formation [10] and thereby the overall changes on their position and orientation must occur prior to the interaction with the DNA. For this reason, our analysis was focused on the recognition helices to characterize the ability of the protein to bind to the DNA.

**Table 2:** Interatomic distances observed in the apo structure, the holo structure, the oxidized average conformation and the reduced average conformation.

	Apo structure (pdb entry 4fx0)	Holo structure (pdb entry 4fx4)	Oxidized average conformation	Reduced average conformation
Chain A	6.2–7.7	6.5–7.4	7.3–8.6	5.9–6.9
Chain B	6.1–8.0	6.1–7.6	7.5–8.3	6.0–7.1

Two values, separated by a hyphen (Å-Å), are displayed for the chain A of each conformation, and other two for the chain B. The first is the intrachain distance between Cys10 and Thr14, and the second is the interchain distance between Cys12 and Asn37’ of the opposite chain.

### The recognition helices

**Fig 2:**
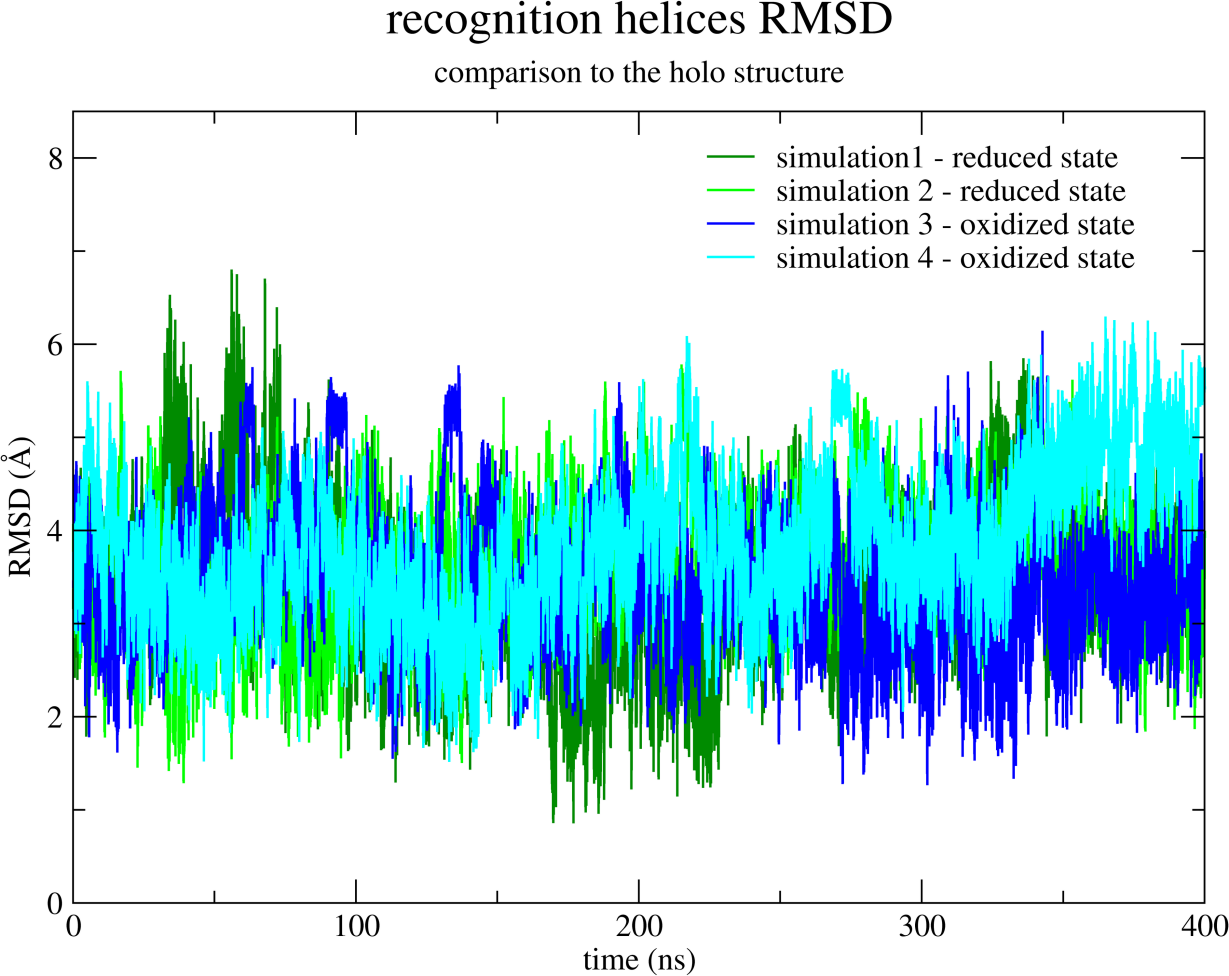
The RMSD between the recognition helices in each simulation and those of the crystallographic structure bound to the DNA (PDB entry 4fx4) is presented as a function of time. Simulation 1 is colored in dark green; simulation 2 is colored in light green; simulation 3 in dark blue and simulation 4 in light blue.

Fig 2 shows the RMSD as a function of time of the recognition helices in each of the four simulations compared to the recognition helices of the crystallographic structure bound to the DNA (PDB entry 4fx4). It is clear that simulation 1 in the reduced state presents a set of conformations which is closer to the crystallographic structure (the minimum RMSD value observed is 0.9 Å). Nevertheless, simulation 3 in the oxidized state also presents conformations that are considerably close to the holo structure (the minimum RMSD value observed is 1.3 Å). Simulation 2 also presents a minimum RMSD value of 1.3 Å, while simulation 4 never shows a RMSD lower than 1.5 Å. This result from the RMSD of the recognition helices is not conclusive and we have made other analyses to obtain information on how much each trajectory approaches the apo and the holo crystallographic structures.

**Fig 3:**
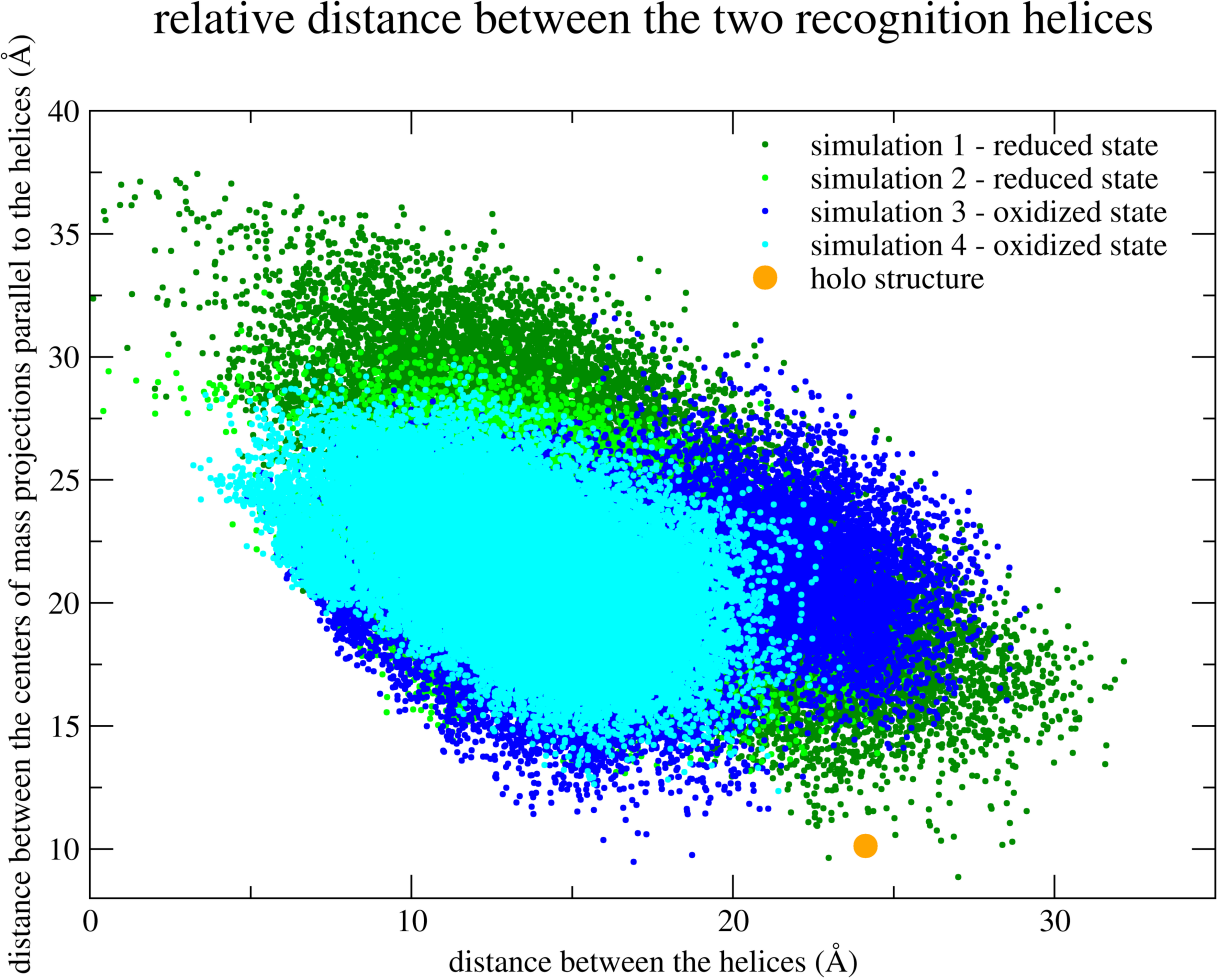
Distribution of the conformations from the four simulations according to the relative orientation of the recognition helices. A dot for each conformation in each of the four simulations is represented differentiating the simulations with different colors (see above). For each dot, the abscissa defines the distance between the two helices which is calculated as the norm of the vector perpendicular to both helices axes. The ordinate defines the distance between the mass centers of both helices projected onto a plane parallel to both helices axes. Simulation 1 is colored in dark green, simulation 2 is colored in light green, simulation 3 in dark blue and simulation 4 in light blue.

Though low RMSD values are certain to represent very similar conformations, the higher the values are, the less information we have on how these differences occur. Are they the result of local deformations or the result of a global change in the helices position? For example, in simulation 1 the conformation with the lowest local RMSD value, when considering only the recognition helices (which is showed in Fig 2) presents a global RMSD value (considering all the α-carbon atoms in the protein) of 1.933 Å compared to the holo structure. Differently, in simulation 2, the conformation with lowest local RMSD value (for the helices) presents a global RMSD value of 2.882 Å. These large difference in the global RMSD values, despite the small differences in the local RMSD values, probably indicate that the analyzed conformation of simulation 1 is globally approaching the holo crystallographic conformation, while the conformation from simulation 2 is the result of only local changes in the recognition helices. With the aim to present a more informative description, we then describe the relative orientation of the recognition helices related to each other by three parameters (see the methods section). The direct dot product between the recognition helices axes do not separate well conformations from simulations 1 and 3, and thereby Fig 3 shows only the other two parameters: the distance between the two helices which is normal to both axes; and the distance between their mass centers which is orthogonal to the direction of the latter. The crystallographic holo conformation is also represented in Fig 3 by an orange circle. In this analysis, it is clear how the conformations of simulation 1 are the ones which are more similar to the crystallographic holo conformation. Both simulations in the reduced state have much higher probability of binding to the DNA than the simulations in the oxidized state. However, the probability of binding to the DNA is low even in simulation 1, so the possibility of an allosteric ligand or another complementary regulating effect cannot be discarded, as previously described [2].

**Fig 4:**
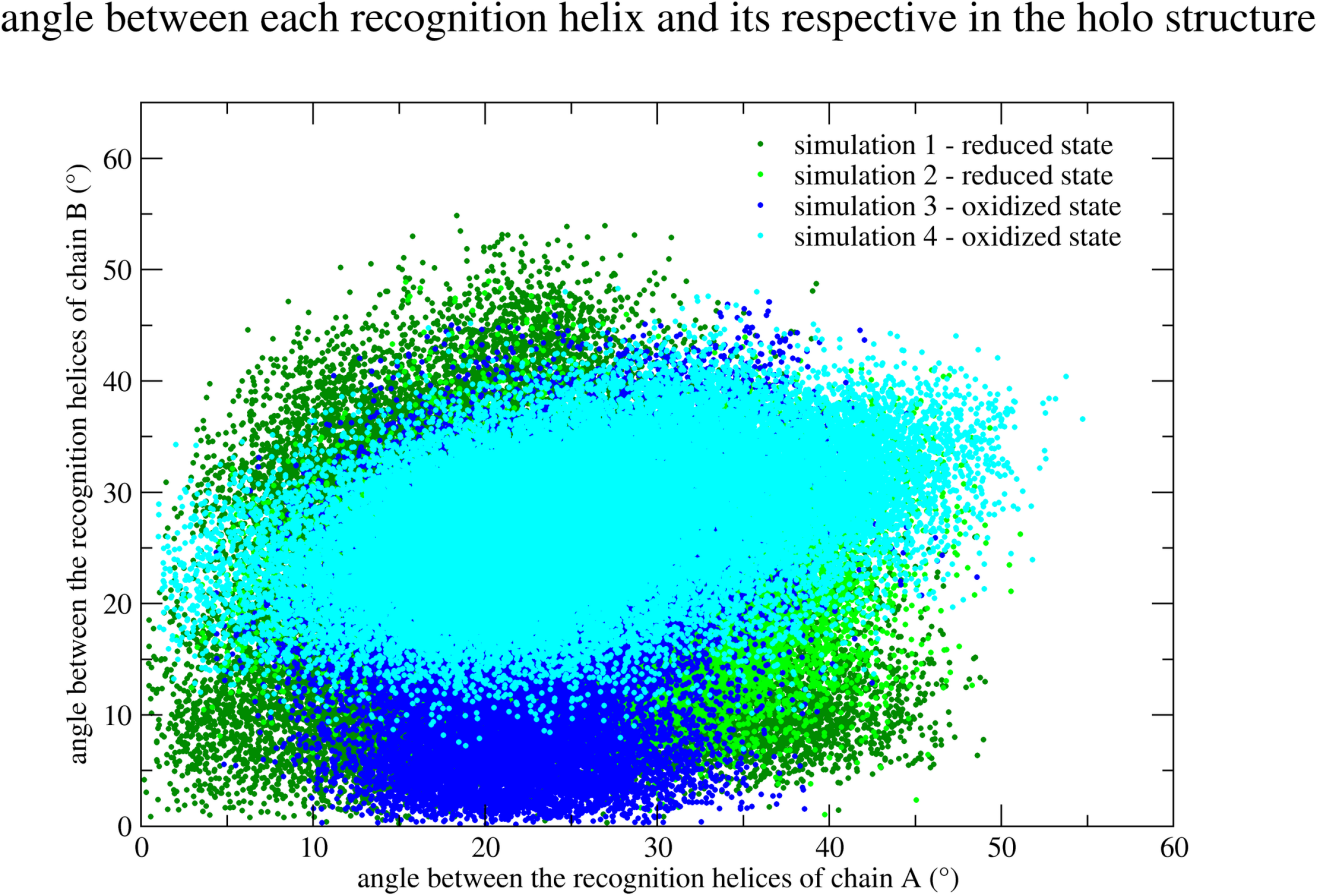
Distribution of the conformations from the four simulations comparing the recognitions helices to the holo crystallographic structure. A dot for each conformation in each of the four simulations is represented differentiating the simulations with different colors (the same color code as in Fig 3). The angle between the axis of each recognition helix and its respective helix of the holo crystallographic structure is represented for both DBDs in the dimer (chain A in the abscissas and chain B in the ordinates). Simulation 1 is colored in dark green, simulation 2 is colored in light green, simulation 3 in dark blue and simulation 4 in light blue. The origin corresponds to the holo crystallographic conformation.

Further descriptions are useful in order to confirm this result. In Fig 4 the conformations in each simulation are analyzed representing a plot of the dot product of each recognition helix and its respective helix in the crystallographic holo structure. Although the RMSD between them may be low in simulation 3 (Fig 2), this result showed in Fig 4 supports the fact that the conformations from simulation 1 are the ones which most resembles the crystallographic holo structure.

**Fig 5:**
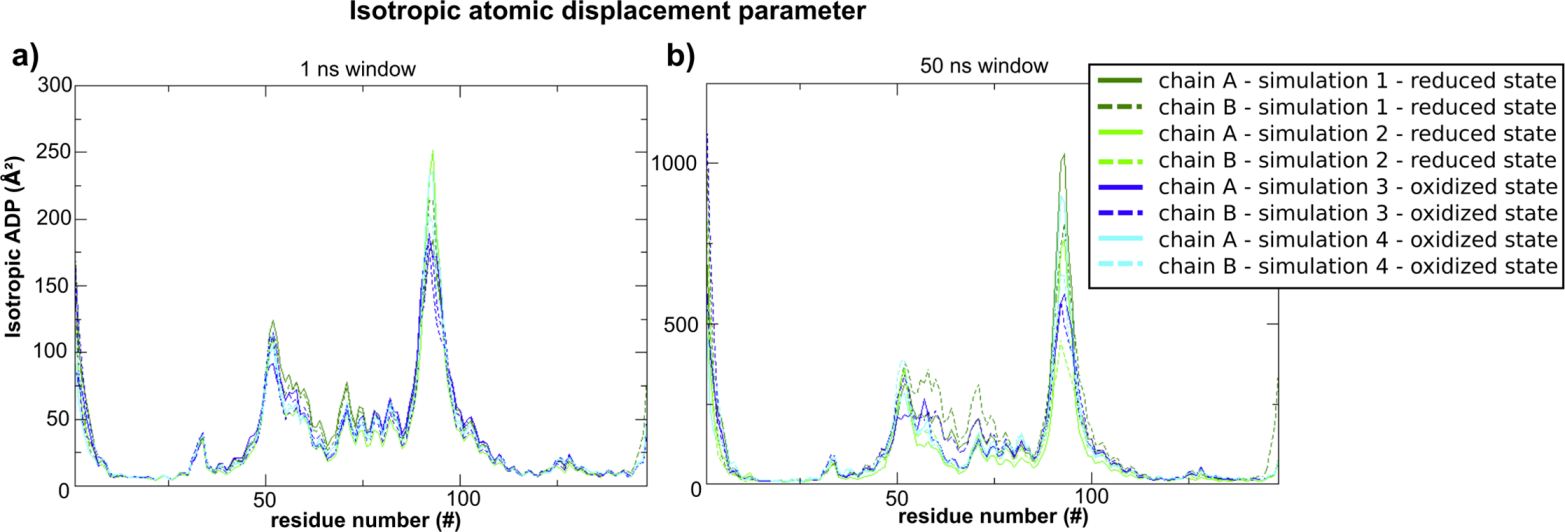
Isotropic ADPs calculated for each simulation and each chain within different time windows. (a) Average values for 1 ns time window; (b) Average values for 50 ns time window. Simulation 1 is colored in dark green, simulation 2 is colored in light green, simulation 3 in dark blue and simulation 4 in light blue.

The conformations in simulation 1 seem also to be more spread out than in the other simulations (see Figs 3 and 4) in the space defined by these selected parameters. Fig 5 presents the comparison of the flexibility of MosR in each simulation represented by the isotropic Atomic Displacement Parameter (isotropic ADP), calculated for time windows of 1 ns and 50 ns. For 1 ns, MosR presents nearly the same flexibility in all simulations. For a 50 ns time window, MosR presents similar flexibility in simulations which share the same initial conformations (simulations 1 and 3 on one side and simulations 2 and 4 on the other). The flexibility of the DBD helices is even higher for the chain B in simulation 1. The different time windows allow distinguishing local from collective motions. Therefore, these results indicate that the variety of orientations of the recognition helices observed in simulation 1 may arise from a collective motion of the DBD in chain B.

This set of results also shows that MosR is more likely to achieve a conformation able to bind to the DNA in a reduced state, since it presents higher flexibility (Fig 5). However, MosR in simulation 2 was also in a reduced state but did not show such capability to approach the DNA-binding orientation of the recognition helices. Thereby, the initial conformation of the N-terminus (in this time-limited simulations) is probably affecting the accessible space of the recognition helices. The next set of results is in regard of this possibility.

### On the N-terminus contribution

**Fig 6:**
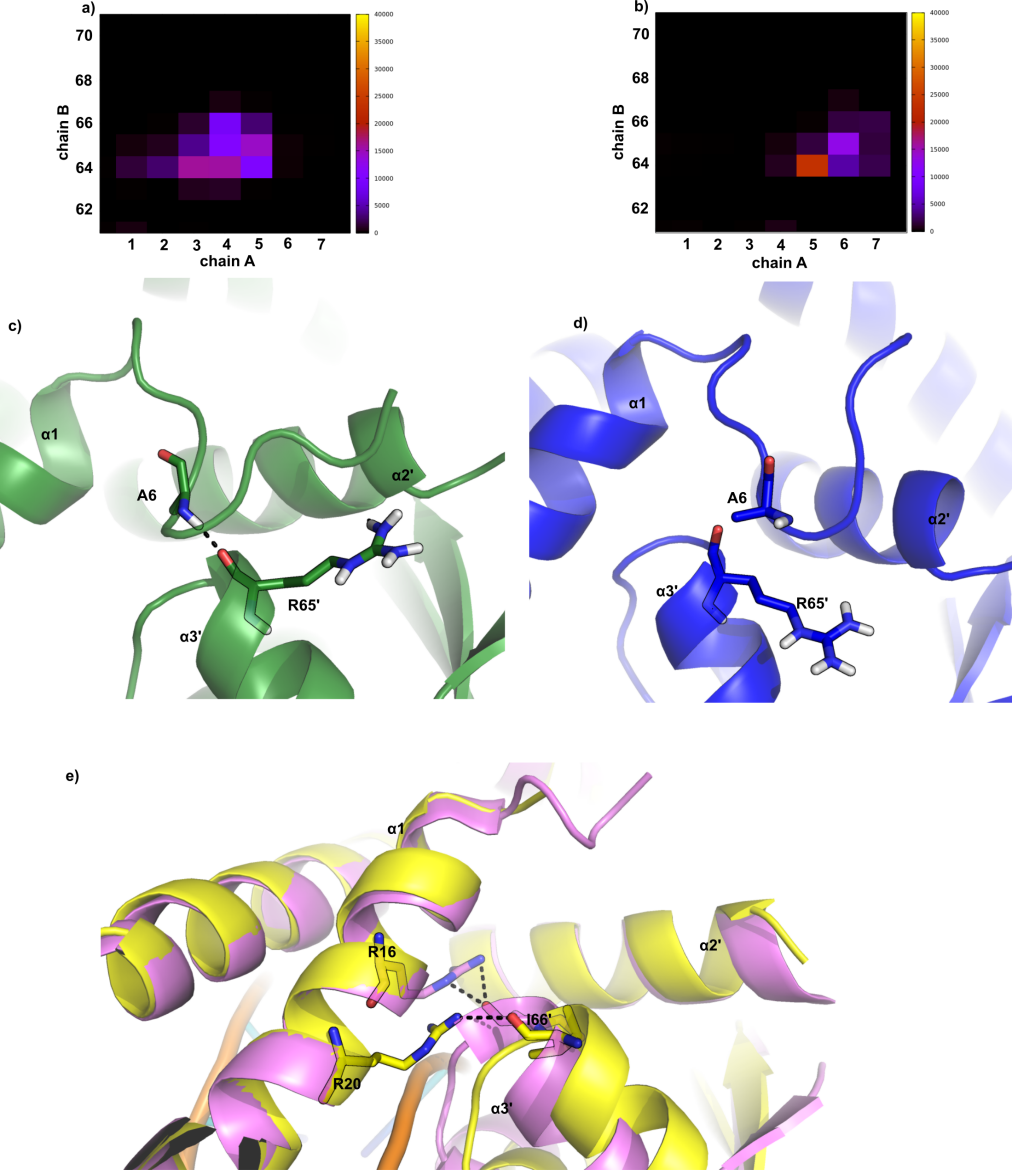
Interactions of the N-terminal with helix α3’ of the opposite chain. (a) and (b) are contact maps showing the number of conformations where a contact was found between residues from 1 to 7 (N-terminal) and residues from 62 to 70 of the opposite chain (helix α3’) during the simulated trajectories. A contact is considered to exist when two α-carbons are not more than 6 Å apart. (a) contact map for the N-terminal of chain A and helix α3’ of chain B from simulation 1, at the reduced state. (b) contact map for the N-terminal of chain A and helix α3’ of chain B from simulation 3, at the oxidized state. (c) a conformation retrieved from simulation 1 highlighting residues Ala6 and Arg65’ drawn in sticks and the hydrogen bond they make in a dashed black line. (d) a conformation retrieved from simulation 3 highlighting residues Ala6 and Arg65’ drawn in sticks. (e) superposition of the holo crystallographic structure in yellow and the apo crystallographic structure in pink highlighting the residues Arg16, Arg20 and Ile66’ drawn in sticks and the hydrogen bond they make in dashed black lines.

The coordinates of the N-terminal residues could not be solved in the crystallographic structures, which indicates that they might be disordered. Thus, one may expect that the N-termini, which should be very flexible, can transiently interact with a broad number of residues in the neighborhood. The pattern of contacts would be similar in all simulations if these interactions were rapidly changing and the simulations were long enough. For example, in all simulations we found that the residue 4 is close to the residues from helix α2’ and the loop that connects to helix α3’ of the other chain, but with slightly different rates. Particularly, we found that the first residues of chain A in simulation 1 and of both chains in simulation 3 are the only ones to interact with helix α3’.This interaction was probably favored by the initial conformation. However, the participating N-terminal residues are slightly different in each simulation, as showed in Fig 6A and 6B. Looking specifically into the polar contacts made by these N-terminal residues and helix α3’, it is clear that the N-terminus from the chain A in simulation 1 is the only one to present an interaction between the nitrogen atom of Ala6 and the oxygen atom from Arg65’ of the chain B. An example of this behavior is the conformations retrieved from simulation 1 and 3, showed in Fig 6C and 6D. In the reduced state (simulation 1) the N-terminal is able to interpose between helix α1 and helix α3’, which does not occur in the oxidized state (simulation 3). The different positions of helix α3’ are in agreement with the experimental data, since the holo crystallographic conformation does show a larger distance between the helix α3’ and the helix α1 than the apo crystallographic structure, as indicated in Fig 6E.

**Fig 7:**
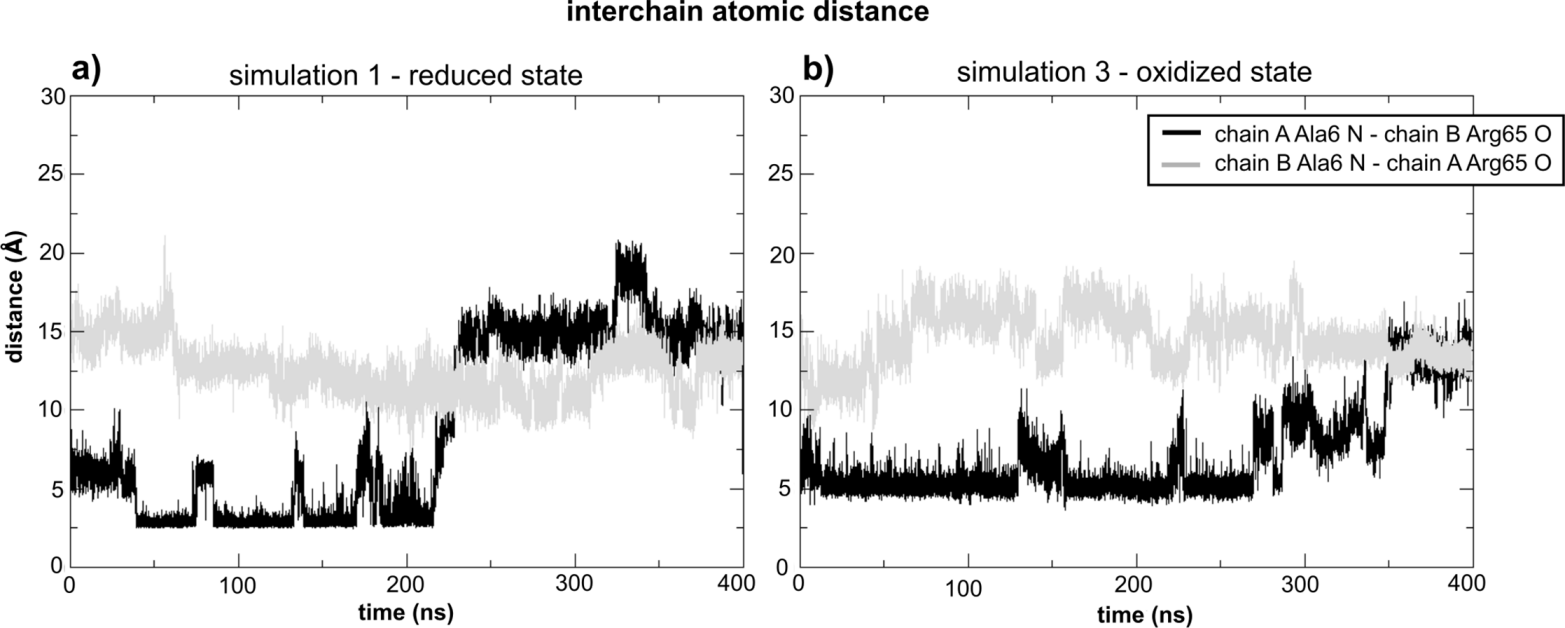
Interchain distance between the nitrogen atom of Ala6 and the oxygen atom of Arg65 of the opposite chain as a function of time. (a) During simulation 1 and (b) during simulation 3.

We have monitored the distance between the nitrogen atom of Ala6 and the oxygen atom from Arg65 in order to verify the behavior of the specific interaction found in simulation 1. Fig 7 shows this distance as a function of time for both N-termini of MosR in simulations 1 and 3. It is clear that this polar interaction is not permanent, but it endures for half of the simulation 1. Coincidently, the least RMSD value of the recognition helices in comparison to the holo crystallographic structure (Fig 2) was found at the first half of simulation 1. On the other hand, this interaction is never formed in simulation 3, although the nitrogen and oxygen atoms maintain a stable distance (around 5 Å) during more than half of the simulation, which indicates an interaction mediated by a water molecule.

**Fig 8:**
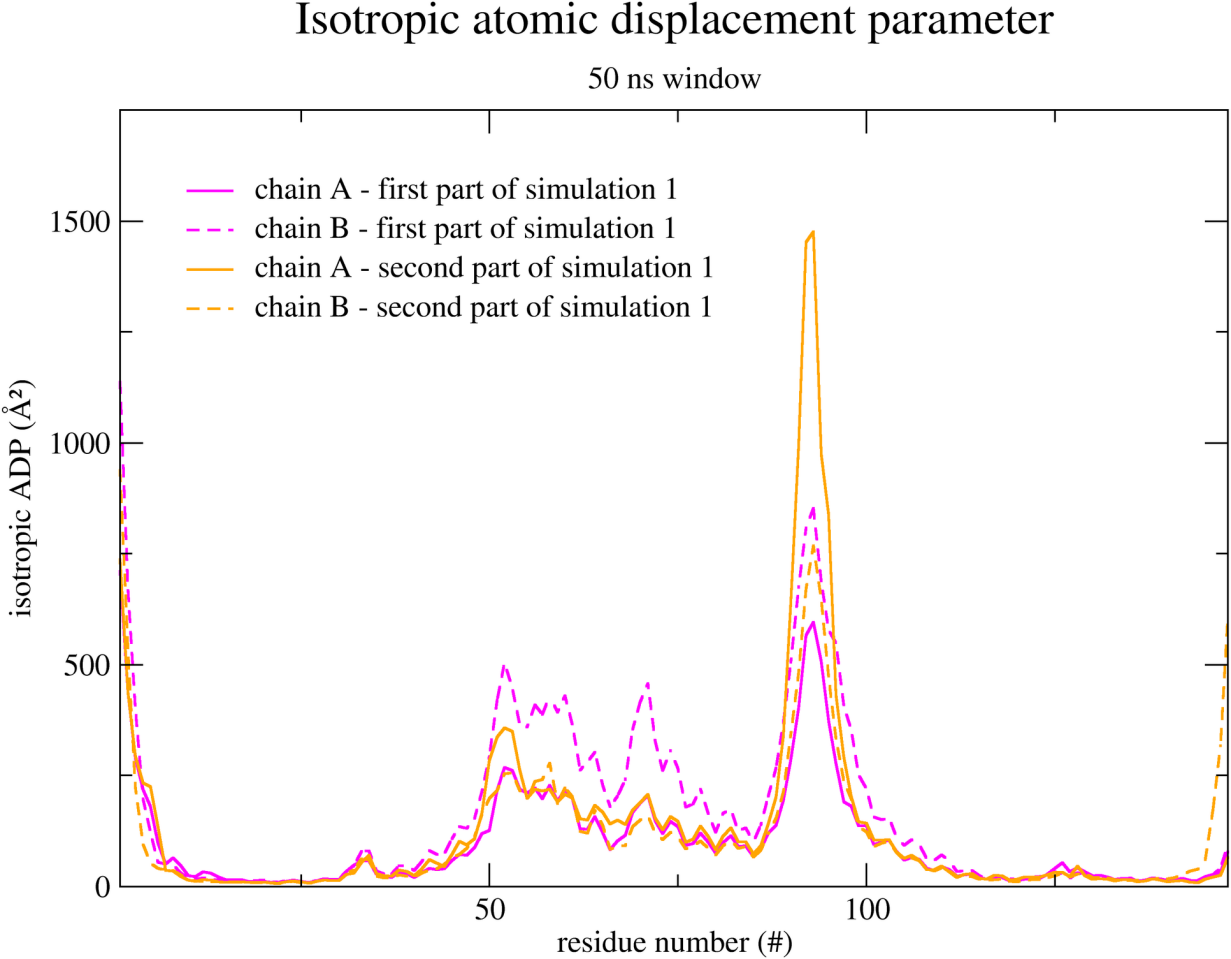
Isotropic atomic displacement parameter calculated within 50 ns time windows and only for the α-carbons. The first half of the simulation 1 is colored in pink, and the second half is colored in orange.

**Fig 9:**
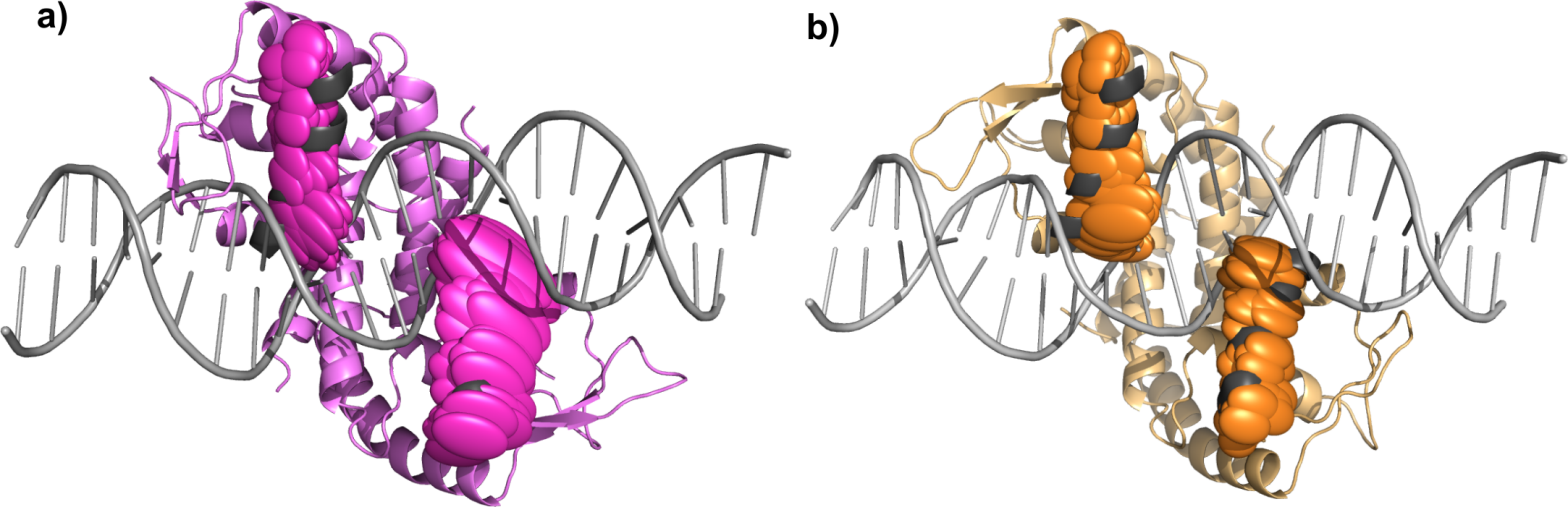
Anisotropic atomic displacement parameter calculated for the first and second halves (200 ns each) of simulation 1, represented by ellipsoids only for the α-carbons. (a) The average conformation of the first half of the simulation 1 is colored in pink, and (b) the average conformation of the second half is colored in orange. The recognition helices of each conformation are superposed to the recognition helices of the holo crystallographic structure (showed in black) while the DNA bound to the holo conformation is colored in grey.

As chain B in simulation 1 was already observed to be the most flexible (Fig 5), we next verified whether the mentioned interaction could be affecting the flexibility of the DBD. Hence we have divided simulation 1 into two halves. Fig 8 shows the calculated isotropic atomic displacement for each part. As observed in this analysis, chain B in the first half is more flexible than in the second half. Moreover, Fig 9 shows the anisotropic atomic displacements calculated for each half of the simulation, which confirms that the higher flexibility of the DBD from chain B is key to allow the best positioning of the recognition helices into the major grooves of the DNA. In short, we observed that the hydrogen bond directly connecting Ala6 in the N-terminal and Arg65’ in the HTH of the opposite chain enhances the flexibility of this DBD.

**Fig 10:**
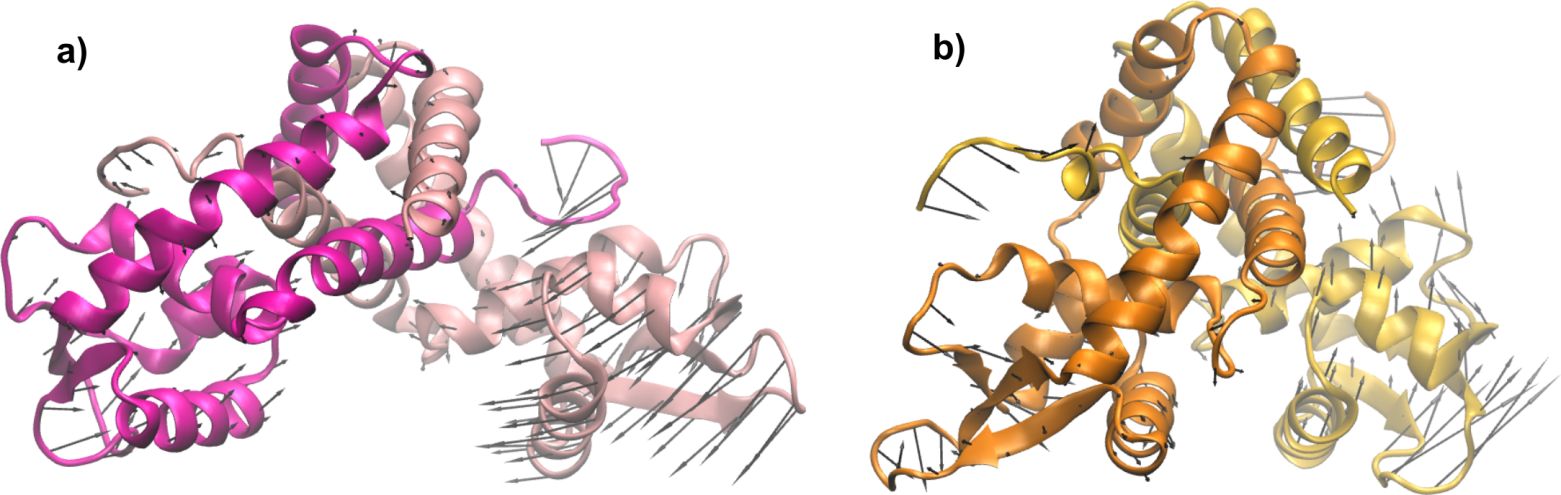
Atomic displacement vectors for each α-carbon representing the harmonic mode with the lowest frequency retrieved. (a) From the first half of simulation 1 and (b) the second half of simulation 1. The conformations showed are the first ones from the periods of analysis. The conformation of the first half of simulation 1 is colored in pink, and the conformation of the second half is colored in orange. Chain B is represented in a lighter color in both conformations.

To further evaluate the differences in the motions performed by MosR during both halves of simulation 1, we have retrieved the motions associated to the lowest frequencies. Fig 10 represents the motion with the lowest frequency of both halves with arrows for each α-carbon, and Table 3 indicates the frequency, amplitude and the degree of collectivity for the five motions with the lowest frequencies (animated movies of the first filtered harmonic modes from the first and the second parts of the simulation 1 can be viewed in S1 and S2 Movies). The harmonic motions retrieved from the first half have higher amplitudes and higher degrees of collectivity than the ones from the second half. It is possible that the increased degree of collectivity arises from a rearrangement of the structure provided by the interaction between Ala6 from the chain A and Arg65 from the chain B. Indeed, the average conformation from this half of the simulation considerably deviates from the average conformation of the second half (Fig 9).

**Table 3:** Frequency, degree of collectivity, global amplitude and local amplitude (only the recognition helices) of the five harmonic modes with the lowest frequencies retrieved from each half of simulation 1.

	First half			Second half		
Frequency (MHz)	Global Amplitude (Å)	Recognition helices amplitude (Å)	Degree of collectivity	Global amplitude (Å)	Recognition helices amplitude (Å)	Degree of collectivity
30.5	66.6	24.8	0.38	60.3	13.6	0.22
61.0	25.3	10.0	0.49	29.4	9.9	0.41
91.5	14.7	5.8	0.48	17.9	3.8	0.22
122.1	10.7	4.8	0.43	17.1	3.3	0.22
152.6	11.1	3.7	0.41	12.9	3.2	0.30

### Imposing a restriction

**Fig 11:**
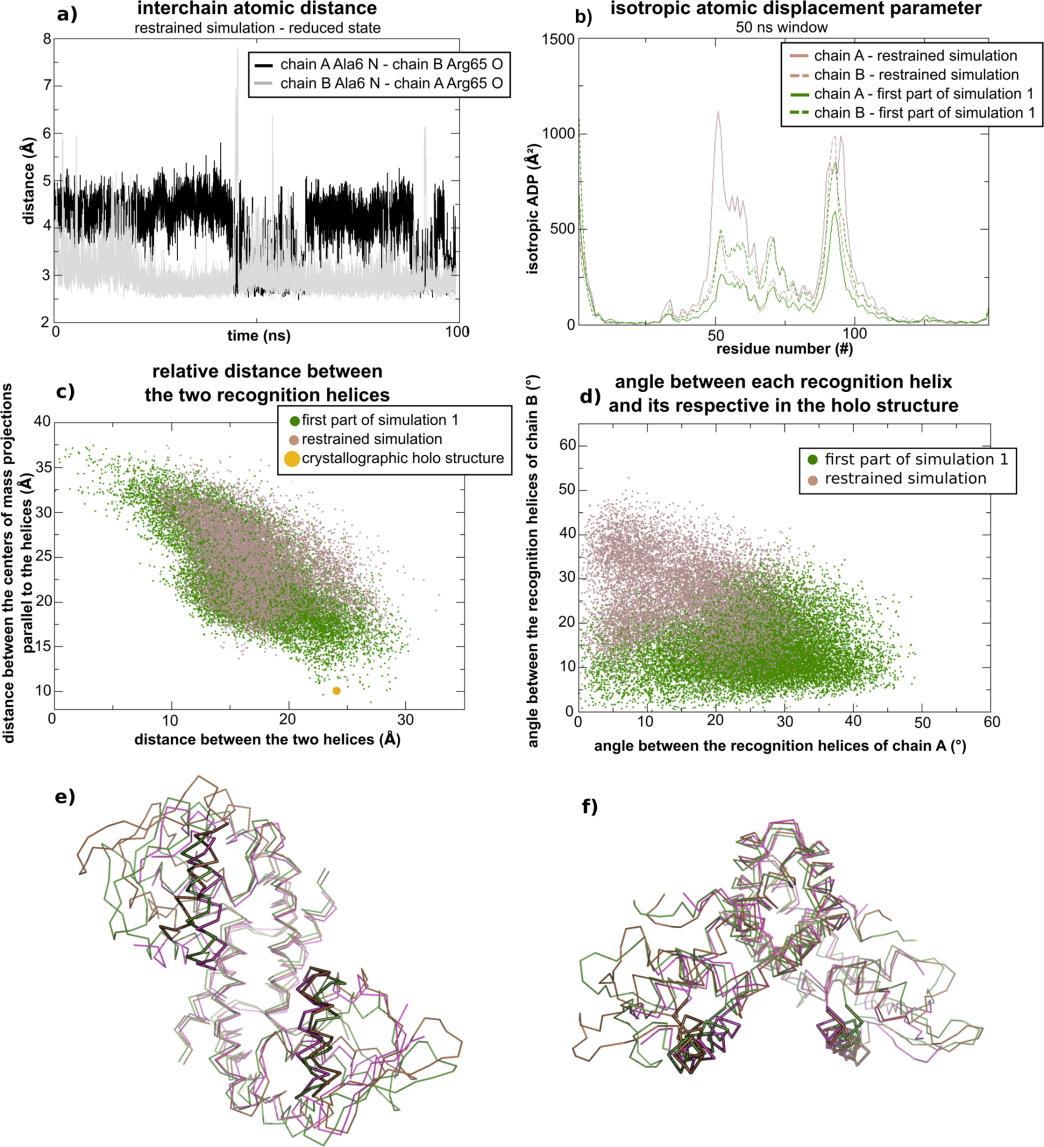
Analyses of the restrained simulation in comparison with the simulation 1, both in the reduced state. Conformations from the restrained simulation are colored in brown, conformations from simulation 1 are colored in green, the holo crystallographic structure is colored in orange, and the apo crystallographic structure in pink. (a) Atomic distances between the nitrogen of Ala6 and the oxygen of Arg65 from opposite chains during the restrained simulation. (b) Isotropic ADPs for both chains of MosR defined by the averages of the 50 ns time windows for the restrained simulation and the first part of simulation1. (c) Relative distance between the two recognition helices. (d) Angle between the axis of each recognition helix and its respective helix of the holo crystallographic structure. (e) and (f) Superposition of the average conformation of the restrained simulation, the average conformation of the first part of simulation 1 and the apo crystallographic structure under two perspectives. The recognition helices are highlighted by a thicker ribbon.

To assure that this specific interaction is in fact associated to an increase of the flexibility and of the affinity for the DNA site, we have simulated a short molecular dynamics in which the distance between the nitrogen atom from Ala6 of one chain and the oxygen atom from Arg65’ of the other chain is restrained. Though the restriction was applied symmetrically for both pair of atoms, only one pair remains close enough to be considered a direct and stable hydrogen bond, as showed in Fig 11A. Fig 11 also shows the isotropic atomic displacements calculated for this new trajectory, as well as the described parameters that indicate the recognition helices approach a conformation similar to the crystallographic holo structure, always comparing with the results obtained for the first half of simulation 1. This set of results shows that chain A from this restrained simulation behaves similarly to chain B in the first half of simulation 1. Thereby, it is possible to associate the detected interaction to the observed differences in the dynamics of MosR that may increase its affinity for the DNA site. Moreover, large conformational changes are also observed when this interaction is present. At last, Fig 11E and 11F show the average conformation from this restrained dynamics and the one from the first half of simulation 1 superposed to the apo crystallographic structure. In the average conformation of the restrained simulation, the recognition helix of chain A is rotated 24.6° from its respective helix in the apo structure, which is compatible with the rotation detected between the apo and holo crystallographic structures. We can also observe that in the average conformations of both simulations only one of the recognition helices remains close to the apo crystallographic structure, while the other presents a rotation approaching the holo conformation. In the restrained simulation, chain A presents a conformational change that not only rotates the recognition helices but also collectively moves the entire DBD, resulting in a RMSD value from the holo crystallographic structure of 1.18 Å. In simulation 1 chain B presents a collective conformational change resulting in a RMSD value from the holo crystallographic structure of 0.94 Å.

## Conclusions

The simulations of MosR showed distinct dynamics of the DBDs depending on the oxidation state of the protein and on the N-terminal interactions. These results not only endorse the known regulatory function of MosR, but also support the conclusions we obtained on the identification and analysis of collective conformational changes and on the role of dynamics in allosteric regulation.

A first conclusion drawn from the simulations is the agreement with some experimental results: The formation of the disulphide bond does promote local changes that mimics real biological characteristics, such as the tension at the beginning of the α-helix in which the cysteines are localized [34] and the redistribution of net charges surrounding the disulphide bridge which in the reduced state is replaced by two protons and two electrons resulting in a polar environment. Moreover, the average conformation calculated from the four simulations of MosR in the absence of the DNA which totalize 1.6 μs, reproduces well the crystallographic apo structure presenting a RMSD value of only 1.0 Å.

It is a more difficult task to determine if a simulated apo conformation is able to bind to DNA by the direct comparison with the holo structure. We have selected three collective variables to describe the position and orientation of the two recognition helices relative to each other. In a space characterized by these variables, conformations of simulation 1 in the reduced state are more spread out than in the other simulations. Hence, some conformations of simulation 1 closely approach the crystallographic holo structure. This broader ensemble is also related to higher flexibility. By calculating the atomic displacements with different time windows, we could attribute this higher flexibility to low frequency motions or collective motions. Moreover, we could specifically identify that only the chain B of MosR in simulation 1 presented this higher flexibility.

The contradictory dynamical behavior of MosR in simulations 1 and 2 indicates that the N-terminal residues are probably affecting the flexibility of the DBD. In fact, counting the contacts made between the N-terminal residues and the opposite DBD showed an interaction unique to the chain B in simulation 1. The interaction made up by the main chain atoms of Ala6 from chain A and Arg65 from the chain B directly connects the N-terminus of the chain A to the helix α3’, the first one in the HTH motif of the chain B. Forcing the proximity of this two residues in a restrained simulation only reinforced the conclusions that this interaction needs to be associated to a higher flexibility of the DBD and its capability to bind to DNA, but intriguing questions arise from these results. How can an interaction, which is a restriction in structure increase the flexibility while it is usually associated to loose and disordered structures? And why is this interaction not observed in the oxidized state?

The studies of Tama and Sanejouand suggest that collective motions are highly subjected to the shape of the protein rather than to more complex atomic properties [33]. Interestingly, the low frequency harmonic modes filtered from the trajectory period where the mentioned interaction appears in the simulation 1 have a higher degree of collectivity than in any other period of the four simulations. The average conformation of the chain B from this particular period deviates 1.39 Å from the apo crystallographic structure (RMSD value) while it deviates only 0.94 Å from the holo crystallographic structure. By allying these RMSD values to the degree of collectivity one may conclude that this period reveals a different shape for MosR, which is closer to the holo structure.

This different shape probably arises from the specific interactions of the N-terminal residues with the helix α3 of opposite chains, but it is primarily originated on the surroundings of the cysteines. Upon the formation of the disulfide bond, not only the beginning of the helix α1 assumes a slightly different conformation, as the Asn37 in the helix α2’ is pushed away from the apolar disulphide bridge. The distancing of the helix α2’ is accompanied by the entire DBD making it impossible to form a hydrogen bond between the main chain atoms of Ala6 and Arg65’. Therefore, the intrachain oxidation of cysteines 10 and 12 is able to preclude the functional conformational change and the associated high flexibility of the neighboring DBD.

A final remark must be made on the analysis method of harmonic mode filtering from the molecular dynamics. It is likely that this method was not thoroughly explored in the early years of 2,000 due to computing limitations, which restricted the molecular dynamic simulations to short times (as compared to hundreds of nanoseconds). However, extending the simulations for longer periods, it does bring a complementary view on the collective motion analysis. Unlike NMA, it does not make approximations on the potential energy landscape and unlikely in essential dynamics, it associates the retrieved modes to the frequency they occur during the simulation. Moreover, motions with the same frequency may be retrieved from different periods of the trajectory, as we have showed here. This method would be of great value to analyze even longer simulations and to obtain a more realistic description on the frequency of the motions with biological relevance.

We conclude that the reduced state of MosR can bind to the DNA and the oxidized state have a much lower probability to achieve this binding. Our simulations show that in the reduced state a subset of conformations with different shape and higher flexibility can be achieved probably favored by the formation of a hydrogen bond between the main chain atoms of Ala6 and Arg65’ of opposite chains. This should generate the correct orientation of the recognition α-helices, able to bind the DNA.

There is still much to learn on the flexibility, frequency and biological functionality of the collective motions, but we expect this work may show a way to further develop these issues.

## Supporting information

S1 Appendix Relative position of the recognition helices. (DOCX)

S2 Appendix The sine filtering analysis. (DOCX)

S1 Movie Filtered harmonic mode number 1 from the first part of the simulation 1. (GIF)

S2 Movie Filtered harmonic mode number 1 from the second part of the simulation 1. (GIF)
