# Supplementary material for: Computer simulations reveal changes in the conformational space of the transcriptional regulator MosR upon the formation of a disulphide bond and in the collective motions that regulate its DNA-binding affinity
Source: PLoS One. 2018 Feb 22;13(2):e0192826. doi: 10.1371/journal.pone.0192826 (PMC5823404; doi:10.1371/journal.pone.0192826)
Supplement: S1 Appendix — (DOCX) [file pone.0192826.s001.docx]

**S1 Appendix: Relative position of the DNA recognition helices**

We selected three parameters to define the position and orientation of the recognition helices relative to each other, one angle and two distances. To calculate them, we first need to characterize an axis for each α-helix, which we considered to be the principal axis of the inertia tensor with the lowest eigenvalue. The inertia tensor and the center of mass are calculated considering only heavy atoms of the main chain of the α-helix.

The angle between the two helices is then the result of the dot product between the versors defining the helices axes. The first distance between the helices is calculated as the norm of the projection of the difference vector of the centers of mass of the helices onto the versor that is normal to both helices axes, which is defined by the cross product of the helices axes. We defined the second distance as the norm of the projection of the difference vector of the centers of mass of the helices onto a plane parallel to both helices axes. Figure A, below, illustrates the three parameters we selected.
