# Supplementary material for: Computer simulations reveal changes in the conformational space of the transcriptional regulator MosR upon the formation of a disulphide bond and in the collective motions that regulate its DNA-binding affinity
Source: PLoS One. 2018 Feb 22;13(2):e0192826. doi: 10.1371/journal.pone.0192826 (PMC5823404; doi:10.1371/journal.pone.0192826)
Supplement: S2 Appendix — (DOCX) [file pone.0192826.s002.docx]

### **S2 Appendix: The sine filtering analysis**

In signal analysis, it is common to use the Fourier transform to filter frequencies [25, 30]. Any discrete and periodical function, *f(t)*, can be represented by a sum of sines and cosines as in equation 1,

(1)

where A_n_ and B_n_ are amplitudes determined by their Fourier transform (equations 2 and 3). Considering, our function, *f(t)*, is the trajectory of a protein, which is represented by a limited number of conformations, sampled by the same interval, *δt*, then *k* is an integer number between 0 and *K*, time t is equivalent to *kδt*, and *K* multiplied by the time interval, *δt*, is equivalent to the total simulated time of the trajectory, *T*. *n* is also an integer number and it stands for the number of the harmonic mode included in the sum, which can be an infinity sum. However, in the numerical treatment for solving the Fourier transform, it is only possible to obtain a limited number of independent modes, which is as much as the amount of provided conformations [25].

(2)

(3)

In signal analysis, a filter for *f(t)* is constructed by selecting a range of frequencies in *A_n_* and *B_n_*, i. e., multiplying its Fourier transform by a modulating function, and transforming back to *f(t)*. This is usually used to remove noisy frequencies, as in image processing.

As showed in [14, 31] this method can be used to filter any time dependent parameter extracted from a molecular dynamics, including the 3N coordinates from the protein trajectory. This last application results in a filtered trajectory, where high or low frequency motions can be neglected and one can analyse the protein motion in certain frequencies of interest.

In [15] this filtering method is used to extract harmonic motions, selecting one specific frequency, and thus compare to the modes obtained by Normal Mode Analysis. The method is applied separately to each one of the 3N coordinates generating new harmonic trajectories, as in equation 4, made up by only one component of the sum in equation 1:

(4)

In equation 4, *Δx_in_* is the displacement of the i^th^ coordinate (of the protein in the 3N space) in the n^th^ harmonic mode and *φ_in_* is the phase. It must be noticed that the phases present in equation 4 depend on *i* (each coordinate) in the same mode *n*, yielding to two amplitudes related to a sine and a cosine to each coordinate *i*. The different phases result in ellipsoidal motions to each atom in a mode and make it difficult to compare the filtered trajectory to other harmonic modes.

This phase problem arises from the periodical boundaries of the trajectory. By imposing zero values on the boundaries for each coordinate and an odd symmetry around *t=0*, the cosine terms are cancelled, and its transform results in an all sine development. This does not imply a loss of generality, since the Fourier transform considers only half a set of sine functions and half a set of cosine functions, while the only sine or cosine transform considers their entire sets [25]. Therefore, all of the three transforms can expand a function to the same number of harmonic functions. It is important to emphasize boundary imposition does not lead to discontinuities, since the Newton equations are symmetric around 0.

To impose the boundary conditions means a function *f(t)*, in the period of T, must obey the equality in equation 5.

(5)

This boundary condition is obtained by subtracting from f(t) a linear function starting at f(0) and ending at *f(T).*

To generate the odd symmetry, we expand the original period of the function to twice its length, from –T to T, [-T,T]. And this other half of the period, [-T,0], is obtained by *f(-t) = -f(t)*.

The sine transform, for a discrete function, is then calculated as in equation 6.

(6)

Since *f(t)* is an odd function, periodic in [–T, T], equation 6 reduces to equation 7 [22].

(7)

Therefore, we only need the data from *t=0* to *t=T*.

As in the filter by Fourier transform [14], after transforming the function to the frequency domain, a range of frequencies, or only one, is filtered by multiplying *F_n_* by a square function *H(n)*.

(8)

Transforming back to the time domain results in a filtered and periodical *f(t)*.

By applying this method to each coordinate from a molecular dynamics trajectory (filtering only one frequency), we obtain a harmonic mode of the simulated protein, described in equation 9,

(9)

Where *F_in_* is the amplitude of the displacement *Δx_in_* of the *i*^th^ coordinate (of the protein in the 3N space) in the *n*^th^ harmonic mode, *ω_in_* is the frequency (which obeys equation 10) and *t* is the actual time. *F_in_* can be written as the product *S_n_ω_in_*, where *S_n_* is a vector in the 3N space.

A summary for the method of sine filtering consists of five topics:

1. Make the initial and final values of the trajectory function equal zero by subtracting a linear function of time that starts at the initial position and ends at the final position of the trajectory.
2. Take the sine transform: This is equivalent to the Fourier transform of a trajectory with a double time period (from –T to T) and anti-symmetric in time.
3. Filter one or a range of frequencies;
4. Transform back to the time domain;
5. Add the linear function subtracted in step 1.

This filtering can be made over any property of the simulation that is a function of time. But when filtering the trajectory of a molecule, these steps should be taken over all of the coordinates separately. The coordinates set may correspond to all the N atoms of the protein, or only a selection of them, as the Cα or a specific region. If so, the amplitude will, likewise, correspond to only this selection, and may also be defined as the magnitude of a vector in a multidimensional Euclidian space. On the other hand, the frequency will not change, as it depends only on the mode number, not on the coordinates. In fact, the frequency depends on the time step and the number of conformations of the trajectory, as in equation 10,

(10)

where *ω_n_* is the frequency, *n* is the mode number, *K* is the total number of conformations and *δt* is the time step between each conformation.
