# Supplementary figures and images for: Computer simulations reveal changes in the conformational space of the transcriptional regulator MosR upon the formation of a disulphide bond and in the collective motions that regulate its DNA-binding affinity
Source: PLoS One. 2018 Feb 22;13(2):e0192826. doi: 10.1371/journal.pone.0192826 (PMC5823404; doi:10.1371/journal.pone.0192826)

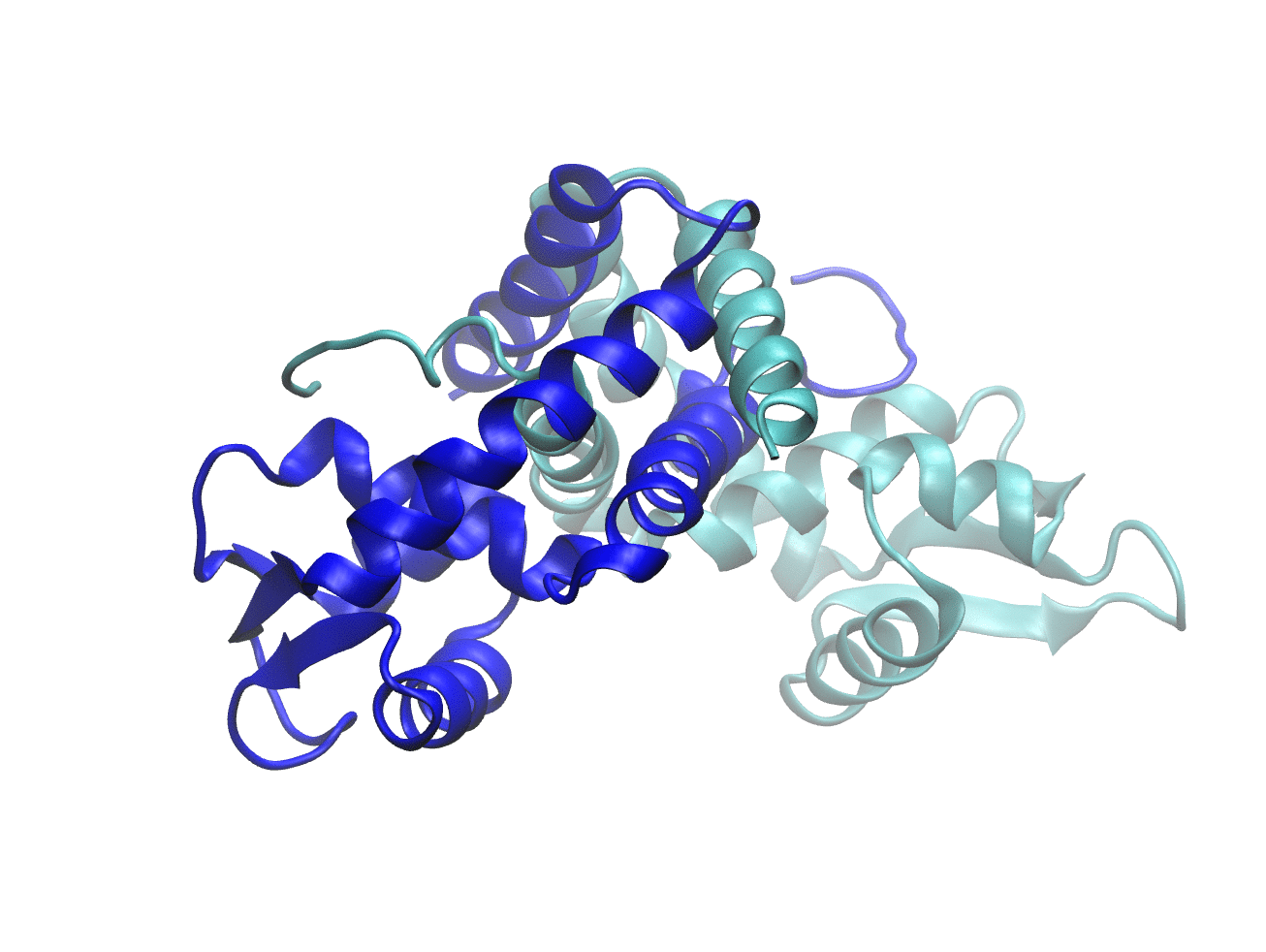

Supplement: S1 Movie — (GIF) [file pone.0192826.s003.gif]

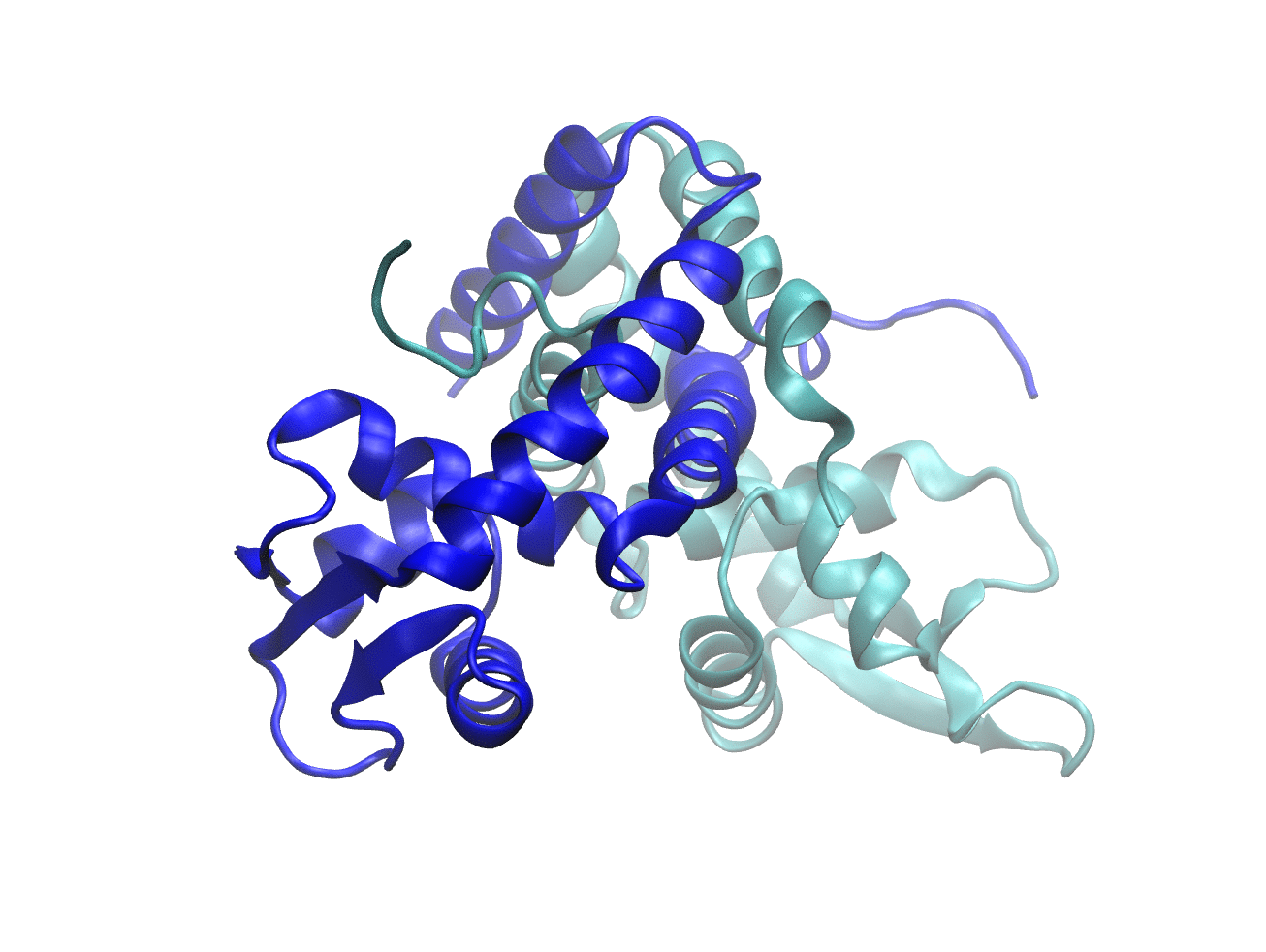

Supplement: S2 Movie — (GIF) [file pone.0192826.s004.gif]
